# Long non‐coding RNA MEG3 inhibits M2 macrophage polarization by activating TRAF6 via microRNA‐223 down‐regulation in viral myocarditis

**DOI:** 10.1111/jcmm.15720

**Published:** 2020-10-13

**Authors:** Yu‐Long Xue, Sheng‐Xiao Zhang, Chao‐Feng Zheng, Yu‐Feng Li, Li‐Hui Zhang, Qin‐Yi Su, Yu‐Fei Hao, Shu Wang, Xue‐Wen Li

**Affiliations:** ^1^ Department of Cardiovascular Medicine Shanxi Dayi Hospital Affiliated to Shanxi Medical University Taiyuan China; ^2^ Department of Rheumatology the Second Hospital of Shanxi Medical University Taiyuan China; ^3^ Department of Genetics Laboratory Linfen Maternity & Child Healthcare Hospital Linfen China; ^4^ Department of Neurology and Stroke Center The First Affiliated Hospital of Jinan University Guangzhou China; ^5^ Department of Rehabilitation Medicine The First Affiliated Hospital of Xiamen University Xiamen China

**Keywords:** inflammation, M1 macrophage polarization, M2 macrophage polarization, MEG3, miR‐223, NF‐κB pathway, TRAF6, viral myocarditis

## Abstract

Viral myocarditis (VMC) commonly triggers heart failure, for which no specific treatments are available. This study aims to explore the specific role of long non‐coding RNA (lncRNA) maternally expressed 3 (MEG3) in VMC. A VMC mouse model was induced by *Coxsackievirus B3* (CVB3). Then, MEG3 and TNF receptor‐associated factor 6 (TRAF6) were silenced and microRNA‐223 (miR‐223) was over‐expressed in the VMC mice, followed by determination of ventricular ejection fraction (LVEF) and left ventricular fractional shortening (LVFS). Dual‐luciferase reporter assay was introduced to test the interaction among MEG3, TRAF6 and miR‐223. Macrophages were isolated from cardiac tissues and bone marrow, and polarization of M1 or M2 macrophages was induced. Then, the expressions of components of NLRP3 inflammatory body (NLRP3, ASC, Caspase‐1), M1 markers (CD86, iNOS and TNF‐α) and M2 markers (CD206, Arginase‐1 and Fizz‐1) were measured following MEG3 silencing. In the VMC mouse model, MEG3 and TRAF6 levels were obviously increased, while miR‐223 expression was significantly reduced. Down‐regulation of MEG3 resulted in the inhibition of TRAF6 by promoting miR‐223. TRAF6 was negatively correlated with miR‐223, but positively correlated with MEG3 expression. Down‐regulations of MEG3 or TRAF6 or up‐regulation of miR‐223 was observed to increase mouse weight, survival rate, LVEF and LVFS, while inhibiting myocarditis and inflammation *via* the NF‐κB pathway inactivation in VMC mice. Down‐regulation of MEG3 decreased M1 macrophage polarization and elevated M2 macrophage polarization by up‐regulating miR‐223. Collectively, down‐regulation of MEG3 leads to the inhibition of inflammation and induces M2 macrophage polarization *via* miR‐223/TRAF6/NF‐κB axis, thus alleviating VMC.

## 1 INTRODUCTION

Myocarditis is an inflammatory disease that occurs in the myocardium, which results in significant alterations in cardiac physiological functions, including reduction in the pumping force of the cardiac muscles and abnormal heart rhythms. The incidence of myocardial inflammation is less than 10% of the population.^1^ However, as the obvious clinical symptoms are not conspicuous at an early stage of myocarditis, the disease often progresses without treatment until the emergence of severe symptoms like chest pain, shortness of breath and body fatigue, which can even progress to chronic heart failure in worse condition.^2^ Etiologies of myocarditis include pathogens, toxins, hypersensitivity and immunologic syndromes, among which viruses such as *Coxsackievirus B3* (CVB3), hepatitis C virus (HCV), human immunodeficiency virus (HIV) and adenovirus have been identified as the prevalent factors.^3^ In particular, CVB3 plays a crucial role in the pathogenesis of viral myocarditis (VMC) and can be used to induce a VMC animal model.^4^, ^5^ Following an infection, if the immune system fails to eliminate a viral infection, autoimmunity can be triggered against the myocardium, which entails the activation of infiltrated macrophages, autoreactive T cells, cytokine and production of cross‐reacting antibodies.^6^, ^7^ Therefore, one can view the pathogenesis of VMC as an inflammatory process. Endo‐myocardial biopsy, in combination with histological, immunological and molecular techniques aids the diagnosis of VMC. Unfortunately, an accurate diagnosis can be difficult to obtain in early disease stages of VMC, when only a few lymphocytic infiltration loci are presented.^6^ Therefore, it is urgent to explore the molecular mechanism underlying VMC, which is beneficial for timely diagnosis and management of VMC.

Non‐coding RNAs, including long non‐coding RNAs (lncRNAs) and microRNAs (miRNAs), play critical roles in maintaining regular physiological processes as well as regulating pathological changes.^8^, ^9^ Maternally expressed gene 3 (MEG3) has been identified to be able to control cancer development,^10^, ^11^ angiogenesis^12^ and differentiation of mesenchymal stem cells.^13^ Indeed, MEG3 has been determined to be an inducer of cardiac fibrosis and diastolic dysfunction, which characterizes cardiac remodelling following cardiac injury.^14^ MEG3 is reportedly associated with and down‐regulation of miR‐223,^15^ which is a heart‐enriched miRNA that protects against CVB3‐mediated VMC damages through its regulation on M1/M2 macrophage polarization.^16^ The imbalance of M1/M2 macrophage polarizations is a key factor in local inflammatory response and tissue repair.^17^ Differential polarization of macrophages has been reported to may generate an opposite inflammatory response.^18^ For example, M1 macrophages could aggravate myocarditis and M2 macrophages could alleviate myocardial inflammation.^19^, ^20^, ^21^ M1 macrophages, which are activated by interferon γ (IFN‐γ) or tumour necrosis factor (TNF), have a proinflammatory action to kill invading microbes. M2 macrophages, which are activated by interleukin (IL)‐4, IL‐10 or IL‐13, play anti‐inflammatory role to reduce autophagy and promote tissue repair.^22^ M2 macrophages have been demonstrated to be related to protection against CVB3‐induced myocarditis.^23^

Moreover, a previous study has shown that the progression of VMC involves tumour necrosis factor receptor‐associated factor number six (TRAF6) and nuclear factor κB (NF‐κB) pathway.^24^ Ge et al reported that TRAF6 promoted lipopolysaccharide (LPS)‐induced inflammatory injury in BV2 mouse microglial cells through the activation of the NF‐κB pathway.^25^ In addition, it has been proven that NF‐κB activation is a pathological event that promotes local inflammation,^26^ which implies the central role of the NF‐κB pathway in VMC. However, the underlying mechanism of TRAF6 and the NF‐κB pathway in regulating VMC remains little understood. In the current study, we explored the roles of MEG3 in the regulation of VMC and found that inhibition of MEG3 can alleviate VMC via up‐regulation of miR‐223 and down‐regulation of the NF‐κB pathway.

## 2 MATERIALS AND METHODS

### 2.1 Ethical statement

All animal experiments were performed in accordance with the *Guide for the Care and Use of Laboratory Animal*. This study was reviewed and approved by the Ethics Committee of Shanxi Dayi Hospital Affiliated to Shanxi Medical University.

### 2.2 Study objects

Male BALB/c mice (n = 105; aged 6 or 7 weeks) were purchased from Hunan Slac Jingda Laboratory Animal Co., Ltd. (Changsha, China). All mice were maintained in a specific pathogen‐free (SPF) room with controlled temperature and humidity under a 12:12 hour light/dark cycle.

### 2.3 Mouse model of VMC

CVB3 was purchased from American type culture collection (ATCC, Manassas, VA, USA), and the 50% tissue culture infectious dose (TCID50) was measured before the infection of HeLa cells. The VMC model was established by intraperitoneally injecting the BALB/c mice with 0.1 mL phosphate‐buffered saline (PBS) containing CVB3 (1 × 10^3^ TCID50). These mice were assigned randomly to several groups, which were subjected to different treatments (15 mice per group): VMC mice were infected with lentivirus expressing short hairpin MEG3 (shMEG3), miR‐223 agomir or shTRAF6. In pre‐experiments, we used three shRNAs to silence MEG3, and the shRNA (sh‐MEG3‐3) with best silencing efficacy under fluorescence microscope was selected for subsequent experiments (Figure S1). The aforementioned lentivirus vectors were purchased from GeneChem (Shanghai, China). Green fluorescent protein (GFP) method was used to detect the expression of lentivirus vector in cells. Therefore, the titre was expressed as ‘gene transfer unit (GTU)/mL’ rather than ‘plaque forming unit (PFU)/mL’. On the first and third day after CVB3 injection, mice were intraperitoneally injected with lentivirus at a dose of 5 × 10^4^ gtu/mouse. The remaining part of the experiment was conducted in accordance with a previously described procedure.^27^ On the seventh day following the viral infection, cardiac tissues or infiltrated macrophages in cardiac tissues were collected. Subsequently, 5 mice in each group were used for histological analysis, and 10 mice were utilized for RT‐qPCR and Western blot analysis.

### 2.4 Isolation and culture of macrophages

Isolation of infiltrated macrophages from cardiac tissues was performed as previously described.^20^ In brief, cardiac tissues were extracted aseptically, and cut into 1 mm^3^ pieces, which were then digested with 0.01% hyaluronidase and 0.1% collagenase II for 2 hours. Then, Ficoll density gradient separation was applied for the isolation of inflammatory cells in infiltrated heart. To harvest macrophages, inflammatory cells were stained with fluorescein isothiocyanate (FITC)‐labelled anti‐F4/80 monoclonal antibody (BD Bioscience, San Jose, CA, USA), followed by isolation using flow cytometry (FACS) (BD Biosciences, San Jose, CA, USA).

Subsequently, the isolation of bone marrow‐derived macrophages (BMDMs) was carried out based on the outline as Bauerfeld described in a previous study.^28^ In brief, femurs and tibias were dissected from normal mice and bone marrow was removed. BMDMs were cultured in Dulbecco's minimal essential medium (DMEM) supplemented with 30% L929‐conditioned medium, 10% fetal bovine serum (FBS) and 2 mmol/L glutamine for 7 days. Next, the cells were cultured in a 6‐well plate with 1 × 10^6^ cells per well. After 1 day in culture, the cells were polarized by RPMI 1640 containing 5% FBS, 10 ng/mL lipopolysaccharide (LPS) and 20 ng/mL IFN‐γ (for M1 macrophage polarization) or 20 ng/mL IL‐4 (for M2 macrophage polarization).

### 2.5 Macrophage transfection

All plasmids were purchased from GenePharma Co., Ltd (Shanghai, China). According to the manufacturer's instructions, BMDMs were transfected using the Lipofectamine 2000 kit (Invitrogen, GenePharma Co., Ltd, Shanghai, China) when cell confluence had reached 70%. After 6 hours of transfection, the medium was changed, and the cells were cultured for 24 hours, followed by 24‐hour macrophage polarization treatment. BMDMs were transfected with plasmids overexpressing or silencing MEG3, miR‐223 mimic or inhibitor, or their relative negative control (NC), alone or in combination in the presence of induced M1 or M2 polarizations.

### 2.6 Transthoracic echocardiography

Following the operator's manufacturer, transthoracic echocardiography was carried out using an ultrasound imaging system (Acuson Sequoia C256, Siemens, Erlangen, Germany) and 13 MHz transducer 7 days after CVB3 infection. Conscious depilated mice were immobilized in a supine position, and the cardiac images were obtained in two‐dimensional mode under a parasternal short‐axis view. The M‐type cursor is positioned perpendicular to the papillary muscle level of the interventricular septum and the posterior wall of the ventricle to obtain the echocardiogram. The left ventricular end‐diastolic diameters (LVEDD), left ventricular end‐systolic diameters (LVESD) and left ventricular end‐diastolic volume (LVEDV) of each mouse was measured 3 times to obtain the mean value. Left ventricular ejection fraction (LVEF) and left ventricular fractional shortening (LVFS) were calculated based on the above parameters using the following formula as an index of cardiac function parameters: LVEF = [(LVEDD − LVEDV)/LVEDV] × 100%; LVFS = [(LVEDD − LVESD)/LVEDD] × 100%.^29^

### 2.7 Morphological analysis

Mice were euthanized by pentobarbital sodium (P3761; Sigma‐Aldrich, St. Louis, MO, USA) at 100 mg/kg 7 days after CVB3 infection. Cardiac tissues were collected, paraffin embedded and cut into sections, which were stained by hematoxylin and eosin (H&E). Myocarditis grading (0‐4) was evaluated as previously described^30^ in a blinded fashion by 2 independent investigators.

### 2.8 Immunohistochemistry (IHC)

The cardiac tissue sections were dewaxed with xylene and rehydrated with gradient ethanol, whereupon incubation was carried out with citrate solution for antigen retrieval with high pressure. Each section was incubated with 50 μL 3% H_2_O_2_ at room temperature for 20 minutes. Then, the sections were incubated with the primary rabbit anti‐TRAF6 antibody (1:1000, ab227560, Abcam Inc, Cambridge, UK) overnight at 4, followed by incubation with 50 μL polymer reinforcing agent for 20 minutes at 37°C and 50 μL enzyme‐labelled anti‐rabbit polymer for 30 minutes at 37°C. Each section was then incubated with 100 μL diaminobenzidine (DAB) for 3‐10 minutes. The specimens were counterstained with hematoxylin, dehydrated with gradient ethanol, mounted with neutral resin and observed under the microscope.

### 2.9 Enzyme‐linked immunosorbent assay (ELISA)

The level of IL‐6, IL‐β and IFN‐γ in supernatant of heart homogenates was determined with the relevant ELISA kit (R&D Systems, Minneapolis, MN, USA) in accordance with the manufacturer's protocol.

### 2.10 Flow cytometry

The single‐cell suspension was stained with FITC‐labelled mouse anti‐CD86 antibody (1:50, #105005, BioLegend, San Diego, CA, USA) or mouse anti‐CD206 antibody (1:50, #141703, BioLegend) and analysed by Fluorescence Activating Cell Sorter (FACS) (BD Biosciences). The data were analysed by FowJo software (Tree Star Inc, Ashland, OR, USA).

### 2.11 Dual‐luciferase reporter assay

The binding site of miR‐223 and TRAF6 was predicted based on the website Targetscan. To verify whether TRAF6 was the target of miR‐223, the artificially synthesized 3′‐untranslated region (3′ UTR) of TRAF6 (TRAF6 WT) was introduced into pMIR‐reporter (Beijing Huayueyang Biotechnology Co., Ltd., Beijing, China). Concurrently, the mutant form in which the potential miR‐223 binding sites were mutated (TRAF6 MUT) was also constructed. The wt‐TRAF6 and mut‐TRAF6 were co‐transfected with miR‐223 mimic or NC‐mimic respectively into HEK‐293T (Shanghai Beinuo Bio Co., Ltd, Shanghai, China). After 48 hours, the cells were lysed, and luciferase activity was determined using luciferase assay kit (K801‐200; Biovision Milpitas, CA, USA) with Glomax20/20 luminometer (Promega, Madison, WI, USA).

### 2.12 RNA isolation and quantitation

The total RNA was extracted from cells using TRIzol (Invitrogen, Carlsbad, CA, USA). Then, the total RNA was reversely transcribed into complementary DNA (cDNA) by TaqMan™ MicroRNA Reverse Transcription Kit (4366596; Thermo Fisher Scientific, Waltham, MA, USA) and High‐Capacity cDNA Reverse Transcription Kit (4368813; Thermo Fisher Scientific). Subsequently, quantitative polymerase chain reaction (qPCR) was conducted on the ABI7500 quantitative PCR reactor (Thermo Fisher Scientific) using SYBR®Premix Ex TaqTM (Tli RNaseH Plus) kit. PCR reaction solution was placed on real‐time fluorescence qPCR (Applied Biosystems, Foster City, CA, USA). With β‐actin and U6 used as internal reference, the relative expression was analysed using the 2^−ΔΔCt^ method. The primers for MEG3, miR‐223, TRAF6, M1 markers (myo‐inositol‐1‐phosphate synthase [iNOS] and TNF‐α), M2 markers (Arginase‐1 and Fizz‐1), and components of the NLR family pyrin domain‐containing 3 (NLRP3) inflammasome (NLRP3, apoptosis‐associated speck‐like protein containing a CRAD [ASC], Caspase‐1) were designed and synthesized by Invitrogen (Table 1).

**TABLE 1:** Primer sequences of RT‐qPCR

Genes	Primer sequence
MEG3	F: 5′‐GGGAGCAGCTATGGATCACC‐3′
	R: 5′‐ATAGCGCCCCCTATTCATGC‐3′
miR‐223	F: 5′‐GCGTGTATTTGACAAGCTGAGTT‐3′
	R: 5′‐GTGTCAGTTTGTCAAATACCCCA‐3′
TRAF6	F: 5′‐ATTCTCGACCAGTCTGAAG‐3′
	R: 5′‐ATGAAGGTTCCCTGTCT‐3′
iNOS	F: 5′‐CCCTTCAATGGTTGGTACATGG‐3′
	R: 5′‐ACATTGATCTCCGTGACAGCC‐3′
TNF‐α	F: 5′‐TCTTCTCATTCCTGCTTGTGG‐3′
	R: 5′‐GGTCTGGGCCATAGAACTGA‐3′
Arginase‐1	F: 5′‐AGACAGCAGAGGAGGTGAAGAGTAC‐3′
	R: 5′‐GGTAGTCAGTCCCTGGCTTATGGT‐3′
Fizz‐1	F: 5′‐CGTGGAGAATAAGGTCAAGGAAC‐3′
	R: 5′‐AGCACACCCAGTAGCAGTCATC‐3′
NLRP3	F: 5′‐AGATGCTGGAATTAGACAACTG‐3′
	R: 5′‐CATTTCACCCAACTGTAGGC‐3′
ASC	F: 5′‐GAAGCTGCTGACAGTGCAAC‐3′
	R: 5′‐TGTGAGCTCCAAGCCATACG‐3′
CAPS1	F: 5′‐ACCACTCGTACACGTCTTGC‐3′
	R: 5′‐TGGGCAGGCAGCAAATTCTT‐3′
U6	F: 5′‐GCTTCGGCAGCACATATACTAAAAT‐3′
	R: 5′‐CGCTTCACGAATTTGCGTGTCAT‐3′
β‐actin	F: 5′‐CATCCGTAAAGACCTCTATGCCAAC‐3′
	R: 5′‐ATGGAGCCACCGATCCACA‐3′

Abbreviations: ASC, apoptosis‐associated speck‐like protein containing a CRAD; iNOS, myo‐inositol‐1‐phosphate synthase; MEG3, maternally expressed 3; miR‐223, microRNA‐223; NLRP3, NLR family pyrin domain‐containing 3; RT‐qPCR, reverse transcription‐quantitative polymerase chain reaction; TNF‐α, tumour necrosis factor‐alpha; TRAF6, TNF receptor‐associated factor 6.

### 2.13 Western blot analysis

Total protein was extracted from tissues or cells using RIPA kit (R0010; Beijing Solarbio Science & Technology Co., Ltd, Beijing, China). The protein concentration was determined by bicinchoninic acid (BCA) protein assay kit (GBCBIO Technologies, Guangzhou, China). Then, 40 µg portions of protein were separated by 10% sodium dodecyl sulphate‐polyacrylamide gel electropherosis (SDS‐PAGE). Subsequently, the proteins were transferred onto polyvinylidene fluoride membrane which was blocked by tris‐buffered saline with Tween 20 (TBST) containing 5% bovine serum albumin (BSA) at room temperature. Next, the membrane was incubated with mouse anti‐GAPDH antibody (1:2000, ab8245), or rabbit antibody against p65 (1:1000, ab16502), phosphorylated p65 (1:2000, ab86299), TRAF6 (1:1000, ab227560), phosphorylated IKBα (1:10 000, ab133462), IKBα (1:1000, ab32518), phosphorylated‐IKKα (1:10 000, ab38515), IKKα (1:10 000, ab32041), phosphorylated IKKβ (1:1000, ab59195) or IKKβ (1:1000, ab124957) overnight at 4°C. Afterwards, the membrane was incubated with horseradish peroxidase (HRP)‐tagged goat anti‐rabbit immunoglobulin G (IgG) secondary antibody (1:1000, ab150077) or goat antimouse IgG secondary antibody (1:2000, ab205719) at room temperature. All the antibodies were purchased from Abcam Inc (Cambridge, UK). After addition of developing solution (NCI4106; Pierce, Rockford, IL, USA), the ratio of the grey value of the target band to that of the reference band analysed by Image J software was calculated.

### 2.14 Fluorescence in situ hybridization (FISH) assay

The subcellular localization of MEG3 was determined in accordance with the instructions of the FISH Tag™ RNA Green Kit (RiboBio Co., Ltd., Guangzhou, China). In brief, cells were inoculated into a 6‐well culture plate and cultured for 1 day. When cell confluence reached 80%, the cells were rinsed with PBS and fixed with 1 mL of 4% polyformaldehyde at room temperature, followed by treatment with protease K (2 μg/mL), glycine and phthalide reagent. Next, 250 μL of pre‐hybridization solution was added into the cells for incubation at 42°C for 1 hour. After removing the pre‐hybridization solution, the cells were subsequently hybridized with 250 μL of hybridization solution containing 300 ng/mL probes at 42°C overnight. Following this, the cells were washed using phosphate‐buffered saline/Tween (PBST) three times, and the cell nucleus was stained using PBST‐diluted 6‐diamidino‐2‐phenylindole (DAPI) solution. After inoculation in a 24‐well plate and staining for 5 minutes, the cells were mounted using anti‐fluorescence quenching agent, followed by fluorescence microscopic observation (Olympus, Tokyo, Japan) with five different visual fields randomly selected.

### 2.15 NF‐κB DNA‐binding activity assay

NF‐kB DNA‐binding activity was determined using Nuclear Extract kit and Trans‐Am NF‐κB/p65 ELISA kit (Active Motif, Carlsbad, CA, USA)^31^ in accordance with the manufacturers’ instruction.

### 2.16 Statistical analysis

The experimental data were analysed with SPSS 21.0 software (IBM Corp., Armonk, NY, USA) and described as mean ± SD. Unpaired *t* test was employed to compare data between two groups, while one‐way analysis of variance (ANOVA) was utilized for testing differences among multiple groups, with Tukey's post hoc test conducted. The comparison of mouse weight at different time point was analysed with repeated measurement ANOVA, followed by Bonferroni post hoc test. Pearson's correlation was applied for correlation analysis. *P < *0.05 was considered as statistically significant.

## 3 RESULTS

### 3.1 MEG3 was highly expressed in VMC mice

**FIGURE 1:**
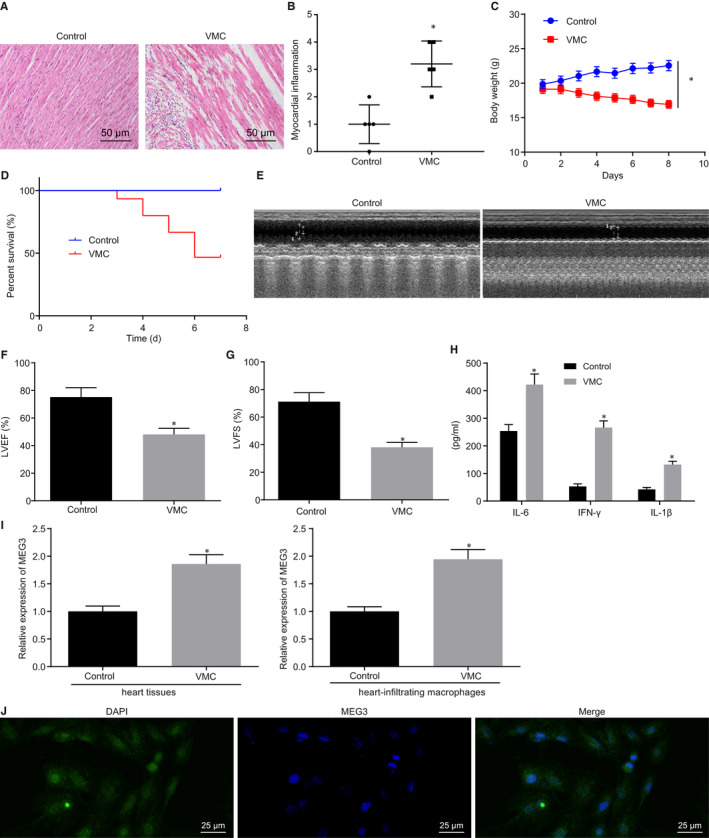
MEG3 was expressed highly in viral myocarditis (VMC) mice. A, H&E staining of myocardial tissues (×200). B, Assessment of myocarditis grading according to results in A. C, Change of mouse body weight. D, Survival rate of mice. E, Echocardiography results of each group. F, Transthoracic echocardiography to determine LVEF. G, Transthoracic echocardiography to examine LVFS. H, Content of IFN‐γ, IL‐6 and IL‐1β in cardiac tissues measured by ELISA. I, MEG3 expression in myocardial tissues and infiltrated macrophages from cardiac tissues on the 7th day after establishment of the VMC model detected by RT‐qPCR. J, Subcellular localization of MEG3 evaluated by FISH assay. *P < 0.05 vs control mice. N = 5 or 10

Firstly, we investigated whether MEG3 was up‐regulated in VMC. After establishing the upon injection of CVB3, pathological changes of myocardial tissues were observed with H&E staining. As depicted in Figure 1A,B, VMC mice presented with severe myocarditis symptoms (Figure 1A,B). Meanwhile, the body weights (Figure 1C, *P* < 0.05) and survival rates (Figure 1D) of VMC mice were significantly reduced. Echocardiography (Figure 1E) showed that LVEF and LVFS of VMC mice were reduced (Figure 1F,G). As shown by ELISA, the contents of IFN‐γ, IL‐6 and IL‐1β in myocardial tissues of VMC mice were markedly increased (Figure 1H). These findings were indicative of successful establishment of VMC mouse model. MEG3 expression was then examined by RT‐qPCR, which showed that MEG3 was expressed at a higher level in myocardial tissues and cardiac infiltrated macrophages of the VMC mice than in normal mice (Figure 1I, *P* < 0.05). Meanwhile, FISH assay was employed to determine the subcellular localization of MEG3, and the results are illustrated in Figure 1J, which revealed that MEG3 expressed both in nuclei and cytoplasm. These data demonstrated that MEG3 expressed at a high level in mice with VMC.

### 3.2 Down‐regulation of MEG3 alleviates myocarditis in mice

**FIGURE 2:**
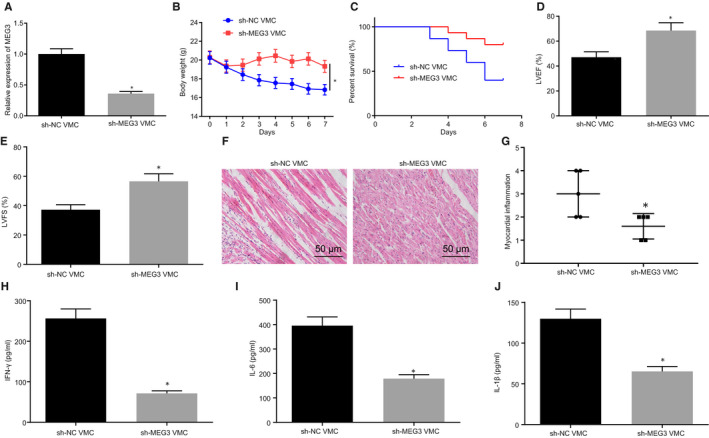
Myocarditis is relieved by decreasing MEG3 in viral myocarditis (VMC) mice. VMC mice were injected with sh‐NC or sh‐MEG3. A, MEG3 expression in myocardial tissues of VMC mice determined by RT‐qPCR. B, Change of mouse body weight. C, Survival rate of mice. D, LVEF determined by transthoracic echocardiography. E, LVFS examined by transthoracic echocardiography. F, H&E staining of myocardial tissues (×200). G, Evaluation of myocarditis according to H&E staining. H/I/J, IFN‐γ, IL‐6 and IL‐1β contents in cardiac tissues monitored by ELISA. The measurement data were described as mean ± SD. Statistical comparisons between two groups were analysed by unpaired *t* test, and repeated measurement ANOVA was used for comparing mice weight at different time point, followed by Bonferroni post hoc test. *P < 0.05 vs VMC mice injected with sh‐NC. N = 5 or 10. The experiment was performed at least three times

To explore the effects of MEG3 on VMC, MEG3 expression was decreased in VMC mice. According to RT‐qPCR, MEG3 expression was conspicuously decreased in VMC mice injected with sh‐MEG3 (Figure 2A). Meanwhile, decreasing the expression of MEG3 resulted in a noticeable increase in body weight (Figure 2B), survival rate (Figure 2C), and LVEF and LVFS (Figure 2D,E) in VMC mice. The results from H&E staining showed that myocarditis in VMC mice was markedly ameliorated following MEG3 down‐regulation (Figure 2F,G). ELISA revealed that silencing MEG3 led to a decrease in IFN‐γ, IL‐6 and IL‐1β contents in myocardial tissues of VMC mice (Figure 2H‐J, *P* < 0.05). These results suggest that MEG3 down‐regulation relieves myocarditis in VMC mice.

### 3.3 MEG3 regulates TRAF6 expression through miR‐223 inhibition

**FIGURE 3:**
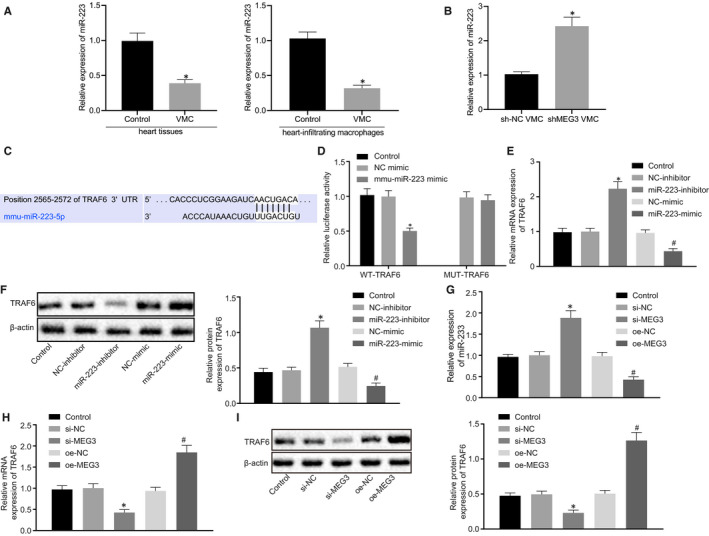
MEG3 decrease miR‐233 expression to up‐regulate TRAF6. A, RT‐qPCR detection of the expression of miR‐223 in myocardial tissues and infiltrated macrophages of viral myocarditis (VMC) mice. B, RT‐qPCR detection of the expression of miR‐223 in MEG3‐silenced VMC mice. C, Binding site between miR‐223 and TRAF6 predicted by Targetscan. D, Targeting relationship between miR‐223 and TRAF6 detected by dual‐luciferase reporter assay. *P < .05 vs NC mimic. E, TRAF6 expression after alteration of miR‐233 level measured by RT‐qPCR. F, TRAF6 expression after alteration of miR‐233 level monitored by Western blot analysis. *P < 0.05 vs treatment with NC‐inhibitor; ^#^ *P* < 0.05 vs treatment with NC‐mimic. G, miR‐223 expression after alteration of MEG3 level examined by RT‐qPCR. H, TRAF6 expression after alteration of MEG3 level measured by RT‐qPCR. I, TRAF6 expression after alteration of MEG3 level monitored by Western blot analysis. *P < 0.05 vs treatment with si‐NC; ^#^ *P* < 0.05 vs treatment with oe‐NC. The measurement data were described as mean ± SD. Comparisons between two groups were made by unpaired *t* test. The experiment was performed at least three times. The control group received no treatment

Previous studies have reported that MEG3 can bind to miR‐223 and inhibit the expression of miR‐223 expression and that miR‐223 is down‐regulated in mice with myocarditis.^15^, ^16^, ^32^ Therefore, we have been suggested that MEG3 might bind to miR‐223 to orchestrate VMC development. In order to verify this hypothesis, miR‐223 expression in myocardial tissue and infiltrated macrophages of VMC mice was detected by RT‐qPCR, and the results (Figure 3A) manifested that miR‐223 expression in VMC mice was significantly lower than that in control mice. Then, miR‐223 expression in VMC mice with silenced MEG3 was determined by RT‐qPCR, which documented (Figure 3B) that compared wi**t**h miR‐223 expression was elevated in VMC mice after MEG3 silencing. Next, we employed Targetscan database (http://www.targetscan.org/) to predict the candidate target genes of miR‐223. Meanwhile, we obtained the GSE19496 data set of sequencing data related to VMC mouse model from the Gene Expression Omnibus (GEO) database (https://www.ncbi.nlm.nih.gov/geo/). Differential analysis was conducted to screen out the significantly up‐regulated genes in VMC, with logFC > 2.5 and *P* < 0.05 as threshold (Figure S2A,B). Then, Venny (v.2.1) tool was used to intersect the genes obtained from these two databases, which finally screened out 19 candidate target genes (Figure S2C). Based on previous investigations, we found TRAF6 could participate in the development of VMC.^33^, ^34^, ^35^ Therefore, we chose TRAF6 as the downstream gene of miR‐223 for further study. By online prediction on Targetscan, a binding site between miR‐223 and TRAF6 was predicted (Figure 3C). Then, with an attempt to verify if TRAF6 was the target gene of miR‐223, the dual‐luciferase reporter assay was carried out (Figure 3D). It was revealed that the luciferase activity of wt‐TRAF6 was reduced by co‐transfection with miR‐223 mimic. However, the luciferase activity of mut‐TRAF6 showed no significant difference between co‐transfection with miR‐223 mimic and NC mimic (*P* > .05). Next, based on RT‐qPCR and Western blot analysis, we found that the mRNA and protein expressions of TRAF6 were significantly decreased by miR‐223 mimic (Figure 3E,F). On the other hand, miR‐223 expression was significantly increased and TRAF6 expression was significantly reduced following treatment with sh‐MEG3, whereas opposite effects were seen after treatment with overexpression (oe)‐MEG3 (Figure 3G‐I). The aforementioned results suggested that MEG3 regulated the expression of TRAF6 through mediating miR‐223.

### 3.4 Decrease of MEG3 alleviates myocarditis via miR‐223 and TRAF6

**FIGURE 4:**
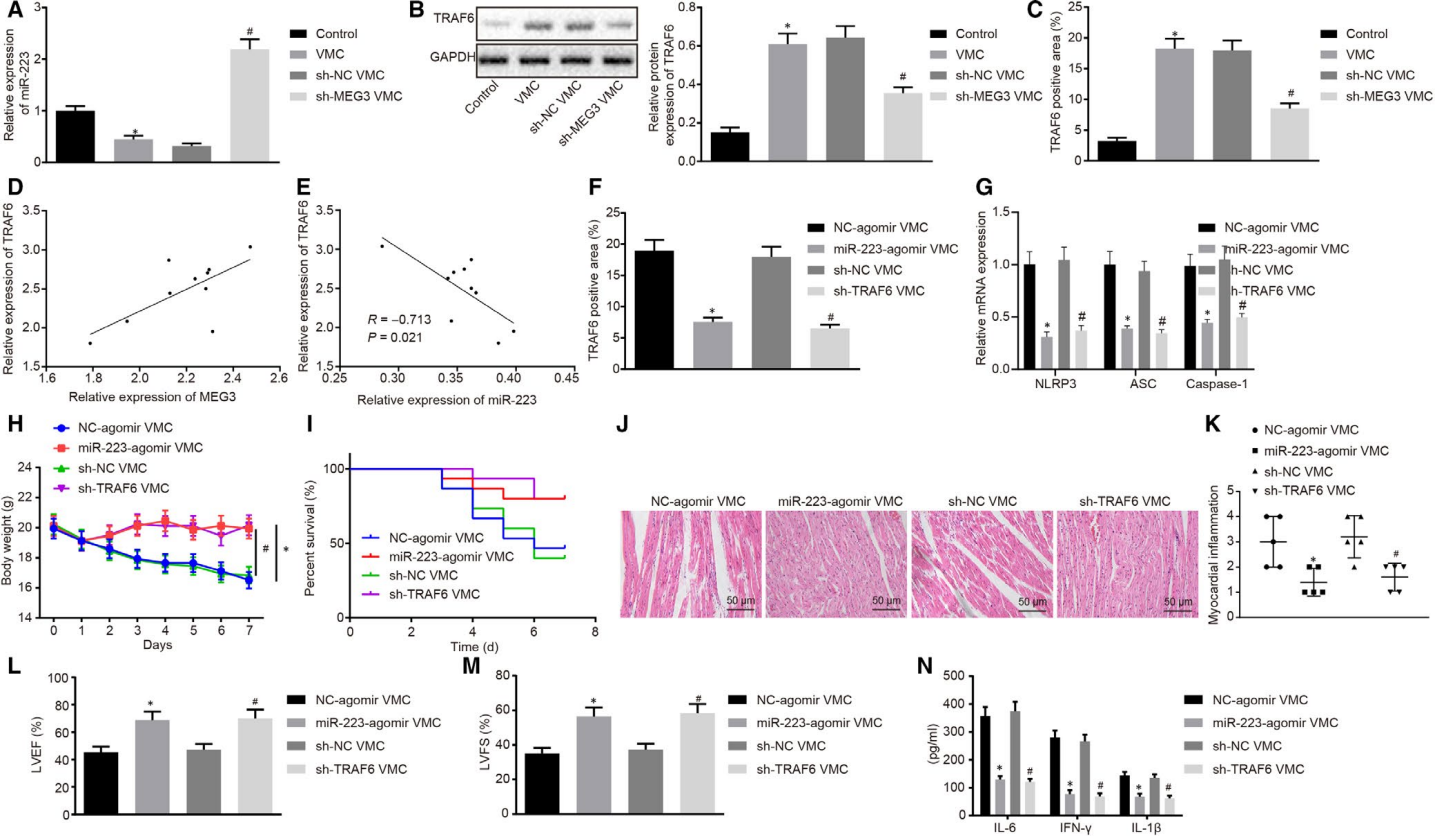
Down‐regulation of MEG3 alleviates myocarditis through inhibition of TRAF6 and increasing miR‐223 expression. A, miR‐223 expression after silencing MEG3 determined by RT‐qPCR. B, TRAF6 expression after silencing MEG3 examined by Western blot analysis. C, TRAF6 expression after silencing MEG3 monitored by IHC (×200). *P < 0.05 vs control mice; ^#^ *P* < 0.05 vs viral myocarditis (VMC) mice injected with sh‐NC. D, Correlation between expression of MEG3 and that of TRAF6 in myocardial tissues from VMC mice analysed by Pearson's correlation. E, Correlation between expression of miR‐223 and expression of TRAF6 in myocardial tissues from VMC mice analysed by Pearson's correlation. F, TRAF6 expression after silencing TRAF6 or over‐expressing miR‐223 monitored by IHC (×200). G, Expression of NLRP3, ASC and Caspase‐1 in cardiac tissues after silencing TRAF6 or over‐expressing miR‐223 detected by RT‐qPCR. H, Change of mice weight after silencing TRAF6 or over‐expressing miR‐223. I, Survival rate of VMC mice after silencing TRAF6 or over‐expressing miR‐223. J, H&E staining of myocardial tissues (×200) after silencing TRAF6 or over‐expressing miR‐223. K, Evaluation of myocarditis after silencing TRAF6 or over‐expressing miR‐223. L, Transthoracic echocardiography to measure LVEF after silencing TRAF6 or over‐expressing miR‐223. M, Transthoracic echocardiography to examine LVFS after silencing TRAF6 or over‐expressing miR‐223. N, IFN‐γ, IL‐6 and IL‐1β contents in myocardial tissues after silencing TRAF6 or over‐expressing miR‐223 tested by ELISA. *P < 0.05 vs VMC mice injected with NC agomir; ^#^ *P* < .005 vs VMC mice injected with sh‐NC. The measurement data were described as mean ± SD. Statistical comparisons between two groups were analysed by unpaired *t* test, and repeated measurement ANOVA was used for comparing mice weight at different time point, followed by Bonferroni post hoc test. N = 5 or 10

Based on the finding that MEG3 regulates TRAF6 by binding to miR‐223, we wished to explore whether miR‐233 and TRAF6 are involved in the mediation of myocarditis by MEG3. Results showed that miR‐223 expression was decreased in VMC mice, which was reversed after MEG3 was down‐regulated (Figure 4A). Western blot analysis and IHC illustrated that the TRAF6 level was increased in VMC mice, which was blocked when MEG3 expression was reduced (Figure 4B,C). According to the Pearson's correlation analysis, TRAF6 expression was found to had a negative correlation with the expression of miR‐223, but was positively correlated with the expression of MEG3 (Figure 4D,E). IHC showed that over‐expressed miR‐223 or down‐regulated TRAF6 resulted in significantly decreased TRAF6 expression (Figure 4F). miR‐223 has previously been shown to decrease the expression of NLRP3 inflammatory corpuscles in the autoimmune myocarditis model, thus promoting the differentiation of dendritic cells (DCs) into tolerant DC phenotypes.^32^ Meanwhile, following miR‐223 up‐regulation or TRAF6 down‐regulation, expression of NLRP3 inflammasome‐related components (NLRP3, ASC and Caspase‐1) was reduced (Figure 4G), mouse body weight was elevated (Figure 4H), survival rate was increased (Figure 4I), myocarditis was inhibited in mice (Figure 4J,K), and LVEF and LVFS rose in VMC mice (Figure 4L,M). ELISA revealed that miR‐223 over‐expression and TRAF6 down‐regulation decreased the contents of IFN‐γ, IL‐6 and IL‐1β in myocardial tissues of VMC mice (Figure 4N). Therefore, down‐regulation of MEG3 alleviated myocarditis by up‐regulating miR‐223 and inhibiting TRAF6.

### 3.5 Down‐regulation of MEG3 inhibits the NF‐κB pathway through miR‐223/TRAF6 axis

**FIGURE 5:**
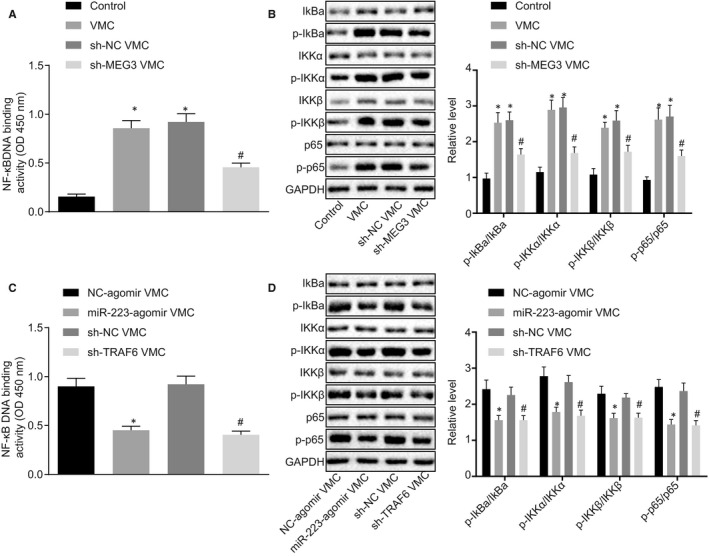
MEG3 down‐regulation suppresses the NF‐κB pathway *via* miR‐223 and TRAF6. A, NF‐κB DNA binding activity after MEG3 was down‐regulated. B, Expression of the NF‐κB pathway components after down‐regulation of MEG3 determined by Western blot analysis. *P < 0.05 vs control mice; ^#^ *P* < 0.05 vs viral myocarditis (VMC) mice injected with sh‐NC. C, NF‐κB DNA binding activity after miR‐223 over‐expression and TRAF6 down‐regulation. D, Expression of the NF‐κB pathway components after miR‐223 over‐expression or TRAF6 knockdown determined by western blot analysis. *P < 0.05 vs VMC mice injected with NC agomir; ^#^ *P* < 0.05 vs VMC mice injected with sh‐NC. All data were presented as measurement data and expressed as mean ± SD. Unpaired *t* test was made in comparisons. N = 5 or 10

To further investigate whether MEG3/miR‐223/TRAF6 affected the NF‐κB pathway, we heuristically examined the activity of the NF‐κB pathway through probing the binding activity of NF‐κB DNA as well as the phosphorylation levels of p65, IKBα, IKKα and IKKβ using western blot analysis. As Figure 5A displayed, NF‐κB DNA binding activity was significantly increased in VMC mice, which was decreased following the down‐regulation of MEG3. Meanwhile, the expression and phosphorylation levels of p65, IKBα, IKKα and IKKβ were increased obviously in VMC mice, which were declined after MEG3 was down‐regulated (Figure 5B). Therefore, down‐regulation of MEG3 could inhibit the NF‐κB pathway in VMC mice. Moreover, it was found that miR‐223 overexpression or TRAF6 knockdown in VMC models led to a significant decline of NF‐κB DNA binding activity (Figure 5C) and an obvious reduce of p65, IKBα, IKKα and IKKβ expression and their phosphorylation (Figure 5D). The aforementioned results led us to conclude that MEG3 down‐regulation inhibits the NF‐κB pathway through miR‐223 over‐expression and TRAF6 inhibition.

### 3.6 Decreasing MEG3 suppresses M1 macrophage polarization but promotes M2 macrophage polarization via miR‐223

**FIGURE 6:**
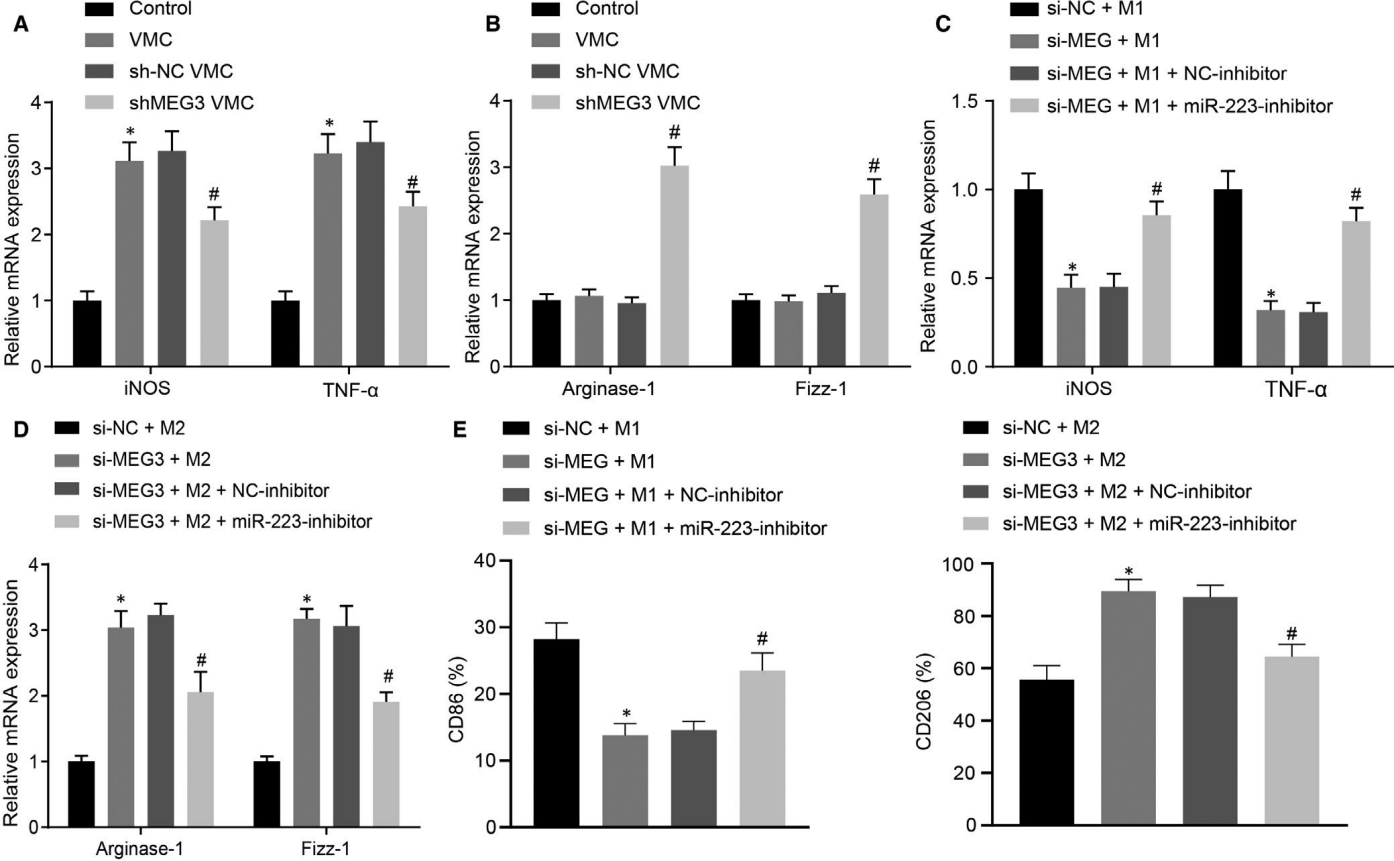
Silencing of MEG3 inhibits M1 macrophage polarization but enhances M2 macrophage polarization via up‐regulating miR‐223. A, Levels of M1 macrophages markers iNOS and TNF‐α in cardiac infiltrated macrophages of viral myocarditis (VMC) mice after down‐regulation of MEG3 determined by RT‐qPCR. B, Levels of M2 macrophages markers Arginase‐1 and Fizz‐1 in cardiac infiltrated macrophages of VMC mice after down‐regulation of MEG3 examined by RT‐qPCR. *P < 0.05 vs control mice; ^#^ *P* < 0.05 vs VMC mice injected with sh‐NC. N = 10. C, Levels of iNOS and TNF‐α after down‐regulation of MEG3 and miR‐223 detected by RT‐qPCR in vitro. D, Levels of Arginase‐1 and Fizz‐1 after down‐regulation of MEG3 and miR‐223 monitored by RT‐qPCR in vitro. E, Levels of CD86 (marker of M1) and CD206 (marker of M2) after down‐regulation of MEG3 and miR‐223 analysed by flow cytometry assay. The measurement data were described as mean ± SD, which was analysed by unpaired *t* test. *P < 0.05 vs the treatment of si‐NC + M1 or M2; ^#^ *P < *0.05 vs the treatment of si‐MEG + M1 or M2 + NC inhibitor. The experiment was repeated 3 times

Prior studies reported that M1 macrophages typically promoted myocarditis and tissue damage through generating proinflammatory cytokines, while M2 macrophages secreted anti‐inflammatory cytokines relevant to tissue repair.^22^, ^36^ Based on a previous study that miR‐223 inhibited M1 macrophage polarization and promoted M2 macrophage polarization,^16^ which led to our hypothesis that MEG3 might mediate macrophage polarization by regulating miR‐223. Arginase‐1, Fizz‐1 and CD206 were shown to be up‐regulated in M2 macrophages while the production of iNOS, TNF‐α and CD86 was specific to M1 macrophages.^37^ Thus, RT‐qPCR and flow cytometry assay were carried out to detect the markers of M1‐polarization (iNOS and TNF‐α) and M2‐polarization (Arginase‐1 and Fizz‐1) as well as CD86 and CD206, respectively. Results showed significantly increased levels of iNOS and TNF‐α in macrophages in heart of VMC mice, which were blocked in response to MEG3 down‐regulation (Figure 6A). At the same time, the levels of Arginase‐1 and Fizz‐1 in macrophages in heart of VMC mice showed no significant difference between VMC mice and normal mice, but were increased after down‐regulation of MEG3 (Figure 6B). Then, to explore whether MEG3 affected macrophage polarization, we isolated primary BMDMs and carried out M1 and M2 macrophage polarization induction experiments in vitro. Results suggested that after induction of M1 macrophage polarization, silencing MEG3 resulted in significant decrease in levels of iNOS and TNF‐α, which was reversed in response to miR‐223 inhibition (Figure 6C). After inducing M2 macrophage polarization was induced, MEG3 knockdown increased the expression of Arginase‐1 and Fizz‐1, which was reversed by down‐regulation of miR‐223 (Figure 6D). Subsequently, results of the flow cytometry assay indicated that when M1 macrophage polarization was induced, CD86 level was declined markedly after MEG3 expression was decreased, which was reversed when miR‐223 was down‐regulated (Figure 6E). Following M2 macrophage polarization induction, the CD206 level was increased when MEG3 was silenced, which was blocked by inhibition of miR‐223 (Figure 6E). In summary, silencing MEG3 inhibits M1 macrophage polarization but boosts M2 macrophage polarization by promoting miR‐223.

## 4 DISCUSSION

Viral myocarditis is a serious heart disease with manifestations ranging from non‐specific systemic symptoms (fever, myalgias, palpitations or exertional dyspnea) to fulminant hemodynamic collapse and sudden death.^3^ Due to the absence of specific guidelines, the current management of VMC is supportive and overall empirical; however, one can hope for the emergence of novel therapies for more effective management of VMC.^38^ Previously, improved ejection function was observed in response to treatment with prednisone or intravenous immune globulin, resulting in significantly elevated LVEF,^39^, ^40^ thus confirming that inflammatory response was critical in VMC progression. Meanwhile, a potential therapeutic role has been reported for miRNAs, cytokines and chemokines on the mechanism of pathogenesis of CVB3‐induced myocarditis have been reported.^41^ In this study, we constructed a VMC model by infecting mice with CVB3, and then showed that inhibition of MEG3 could suppress the NF‐κB pathway as well as increase M2 macrophage transition through miR‐223‐mediated down‐regulation of TRAF6, therefore, ameliorating myocarditis in the VMC mice.

Firstly, VMC cardiac tissues were found to have high MEG3 expression. Previous work has shown a few lncRNAs have been determined to be involved with cardiac inflammation. For instance, TUG1 can bind to miR‐29b and lead to the suppression of cellular apoptosis and inflammatory response in H9c2 cardiomyoblasts treated with LPS.^42^ MALAT1 can regulate the p38 MAPK/NF‐κB pathway by regulating miR‐125b, which aggravates cardiac inflammation and dysfunction induced by sepsis.^43^ One plasma GAS5 has been applied as a novel biomarker for coronary artery disease.^44^ Hence, down‐regulation of certain lncRNAs seemingly plays an anti‐inflammatory role, which could potentially guide the design of a new treatment option for VMC.

Secondly, we find that MEG3 regulates TRAF6 by binding to miR‐223. Numerous miRNAs have been identified as regulators for VMC. For example, overexpression of miR‐1 can promote VMC progression *via* Cx43 suppression.^44^ miR‐223‐3p alleviated experimental autoimmune myocarditis by repressing NLRP3 inflammasome expression and promoting the polarization of DCs towards a tolerant DC phenotype.^32^ miR‐223 is a circulating miRNA that involves in the inflammatory process of hepatitis and other chronic diseases.^45^ A previous study reported that miR‐223/Pknox1 axis can control macrophage polarization, protecting mice from CVB3‐caused VMC.^16^ Hence, miR‐223 acts as an anti‐inflammatory factor in virus‐induced myocarditis. However, in LPS‐treated H9c2 cardiomyoblasts, emodin can alleviate cell injury through the down‐regulation of miR‐223. Therefore, miR‐223 plays a pro‐inflammatory factor in LPS‐induced myocarditis. There is abundant evidence that LPS is a type of TLR ligand, which selectively stimulates and generates classically activated M1 macrophages. When the inflammatory response progresses, IL‐4, produced by type 2 T helper cells, stimulates M2 macrophage generation,^46^ which antagonizes the M1 effect to produce a net reduction in inflammation that is conducive to recovery from injury. Based on the aforementioned results, we concluded that the seemingly contradictory role of miR‐223 in myocarditis is explicable by distinct inducers for myocarditis as well as effects of miR‐233c on different regulatory pathway.

Finally, inhibition of MEG3 can inactivate the NF‐κB pathway and promote M2 macrophage polarization. The NF‐κB pathway is a conserved pathway that induces a stress reaction, alerting host defence against microbe invasion. The primary inflammatory signals can activate expression of inflammation‐related factors through NF‐κB mediators such as IKK, p50, p65 and p105,^26^ which expands inflammatory responses and induces cell apoptosis and autophagy, aggravating tissue injury. In addition to MEG3, several other oncogenic lncRNAs have also been determined to regulate the NF‐κB pathway, including lncRNA HOX transcript antisense RNA (HOTAIR) promoting TNF‐α production in LPS‐treated cardiomyocytes^47^ and lncRNA H19 promoting atherosclerosis mediated by MAPK.^47^ M1/M2 macrophage homeostasis controls the net inflammatory response, in which type M2 is an antagonist that reduces proinflammatory factors. The NF‐κB pathway regulates the M1/M2 macrophage balance to participate in inflammation. Phosphorylation of IKK protein is increased in response to the activation of TLR4 ligand such as LPS, which further promotes the transcriptional activity of p65 and p50. p65/p50 increases the expression of iNOS and HIF1α, subsequently resulting in elevated production of NO radical, which is the effector of M1 macrophages. The inhibition of the NF‐κB pathway can suppress M1 populations.^48^ The effect generated by M2 macrophages is activated by IL‐4 or IL‐13 and mediated by transcriptional factor STAT6 protein. These results suggest that the NF‐κB pathway regulates M2 macrophage generation by regulating M1/M2 homeostasis. Nevertheless, there is still insufficient evidence that NF‐κB‐related factors directly interact with M2 macrophage is still insufficient. Therefore, the NF‐κB pathway might regulate M2 macrophage generation in VMC through its regulatory role on M1/M2 homeostasis.

## 5 CONCLUSIONS AND PERSPECTIVE

**FIGURE 7:**
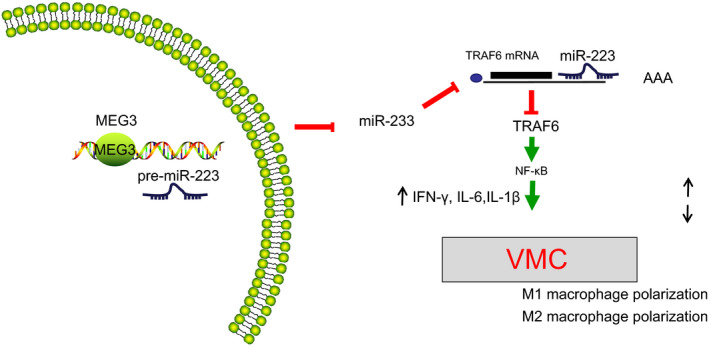
Molecular mechanism of MEG3 regulating viral myocarditis (VMC) *via* the miR‐223/TRAF6/NF‐κB axis. Down‐regulation of MEG3 up‐regulates miR‐223, thus inhibiting TRAF6, inactivating the NF‐κB pathway and blocking the production of IFN‐γ, IL‐6 and IL‐1β, which inhibits polarization of M1 macrophages and boosts polarization of M2 macrophages, therefore, ameliorating myocarditis in VMC mice

Taken together, our findings revealed that MEG3 could participate in the regulation of homeostasis in M1/M2 macrophage. A high expression of MEG3 was observed in the cardiac tissues obtained from VMC mice, and MEG3 was capable of regulating TRAF6 *via* binding to miR‐233. Furthermore, inhibition of MEG3 could result in down‐regulation of the NF‐κB pathway, thus reducing M1 type macrophage and increasing M2 macrophage polarization, which might reduce cardiomyocyte injury (Figure 7). Therefore, the MEG3/miR‐233/TRAF6/NF‐κB axis should be considered as a promising therapeutic target for VMC.

## CONFLICT OF INTEREST

The authors declare that they have no conflict of interest.

## AUTHOR CONTRIBUTION

**Yu‐Long Xue:** Conceptualization (equal); data curation (equal); investigation (equal); project administration (equal); software (equal); writing – review and editing (equal). **Sheng‐Xiao Zhang:** Conceptualization (equal); data curation (equal); formal analysis (equal); investigation (equal); methodology (equal); writing‐original draft (equal). **Chao‐Feng Zheng:** Conceptualization (equal); data curation (equal); formal analysis (equal); investigation (equal); writing – review and editing (equal). **Yu‐Feng Li:** Methodology (equal); resources (equal); software (equal); writing‐original draft (equal). **Li‐Hui Zhang:** Resources (equal); visualization (equal); writing‐original draft (equal). **Qin‐Yi Su:** Resources (equal); validation (equal); writing – review and editing (equal). **Yu‐Fei Hao:** Visualization (equal); writing‐original draft (equal); writing – review and editing (equal). **Shu Wang:** Writing‐original draft (equal); writing – review and editing (equal). **Xue‐Wen Li:** Supervision (equal); writing‐original draft (equal); writing – review and editing (equal).

## Supporting information

Fig S1

Fig S2

## Data Availability

The data sets used and/or analysed during the current study are available from the corresponding author on reasonable request.
